# Influence of Supplemental Dietary Cholesterol on Growth Performance, Indices of Stress, Fillet Pigmentation, and Upper Thermal Tolerance of Female Triploid Atlantic Salmon (*Salmo salar*)

**DOI:** 10.1155/2022/6336060

**Published:** 2022-10-28

**Authors:** Eric H. Ignatz, Rebeccah M. Sandrelli, Sean M. Tibbetts, Stefanie M. Colombo, Fábio S. Zanuzzo, Ashley M. Loveless, Christopher C. Parrish, Matthew L. Rise, A. Kurt Gamperl

**Affiliations:** ^1^Department of Ocean Sciences, Memorial University of Newfoundland and Labrador, St. John's, NL, Canada A1C 5S7; ^2^National Research Council of Canada, Aquatic and Crop Resource Development Research Centre, Halifax, NS, Canada B3H 3Z1; ^3^Department of Animal Science and Aquaculture, Dalhousie University, Truro, NS, Canada B2N 5E3

## Abstract

The salmon aquaculture industry must be proactive at developing mitigation tools/strategies to offset the potential negative impacts of climate change. Therefore, this study examined if additional dietary cholesterol could enhance salmon production at elevated temperatures. We hypothesized that supplemental cholesterol could aid in maintaining cell rigidity, reducing stress and the need to mobilize astaxanthin muscle stores, and improving salmon growth and survival at high rearing temperatures. Accordingly, postsmolt female triploid salmon were exposed to an incremental temperature challenge (+0.2°C day^−1^) to mimic conditions that they experience in sea cages in the summer, with temperature held at both 16 and 18°C for several weeks [i.e., 3 weeks at 16°C, followed by an increase at 0.2°C day^−1^ to 18°C (10 days), then 5 weeks at 18°C] to prolong their exposure to elevated temperatures. From 16°C onwards, the fish were fed either a control diet, or one of two nutritionally equivalent experimental diets containing supplemental cholesterol [+1.30%, experimental diet ^#^1 (ED1); or +1.76%, experimental diet ^#^2 (ED2)]. Adding cholesterol to the diet did not affect the salmon's incremental thermal maximum (IT_Max_), growth, plasma cortisol, or liver stress-related transcript expression. However, ED2 appeared to have a small negative impact on survival, and both ED1 and ED2 reduced fillet “bleaching” above 18°C as measured using SalmoFan™ scores. Although the current results suggest that supplementing salmon diets with cholesterol would have few/minimal benefits for the industry, ≤ 5% of the female triploid Atlantic salmon used in this study irrespective of diet died before temperature reached 22°C. These latter data suggest that it is possible to produce all female populations of reproductively sterile salmon that can withstand summer temperatures in Atlantic Canada.

## 1. Introduction

With the stagnation of the world's capture fisheries, the aquaculture industry now provides almost half of the world's seafood [1]. However, with annual global seafood demand expected to increase by 47 million tonnes by the early 2020s, expansion of the aquaculture sector at current rates is only projected to meet 40% of this requirement [2]. Anthropogenic-induced climate change will only exacerbate this situation, as increases in water temperature and acidification, and low oxygen levels (hypoxia), are all anticipated to have long-term impacts on marine aquaculture production [3–5]. For example, the south coast of Newfoundland experienced an unprecedented heat wave in the summer/fall of 2019 that resulted in the loss of more than 2.6 million Atlantic salmon [6]; and Australian (Tasmanian) cage-sites are already experiencing temperatures as high as 23°C in combination with severe hypoxia [7], and such conditions are negatively affecting salmon production [8]. There are several strategies that could potentially mitigate (reduce) the effects of climate change on salmon aquaculture production, such as selective breeding, the use of new genetic strains, changes in farm management/fish husbandry practices (i.e., improving site selection and/or the use of deeper nets), and altering feed formulations [4, 9–11].

This project addressed the latter of these options by examining if inclusion of higher levels of dietary cholesterol could enhance salmon growth performance and thermal tolerance, as well as reduce stress and the loss of fillet pigmentation (colour) at high temperatures [8, 12, 13]. This approach was taken because, while Irvine et al. [14] showed that increased dietary cholesterol improved the survival of goldfish (*Carassius auratus*) at high temperatures; no such research has been conducted with farmed salmonids. Cholesterol is an important component of cellular membranes and critical for maintaining membrane fluidity/rigidity as environmental temperatures increase [15–19]. Furthermore, in Tasmania, high temperatures at Atlantic salmon sea cage sites resulted in a decrease in fillet colouration (i.e., “bleaching”: [8, 12, 13]). Astaxanthin, a carotenoid typically added to salmon diets to enhance fillet pigmentation, is the most expensive ingredient (per kg) in salmon feed [20], and therefore, producers need to maximize its deposition and retention in the fish's muscle (fillet). Increasing dietary levels of cholesterol may assist with this, as cholesterol has been shown to increase astaxanthin plasma transport in Atlantic salmon and may enhance its subsequent retention in the fillet [21, 22]. In addition, there are some studies that report other positive effects of increased dietary cholesterol levels. Dietary cholesterol supplementation has been shown to improve the rainbow trout's (*Oncorhynchus mykiss*) immune response and disease resistance to the bacterial pathogen *Aeromonas hydrophila* [23]. Changes in the sterol : phospholipid ratio have been reported to be a key response to temperature in salmonid liver membranes [24]. Further, cardiac performance/function is a key determinant of fish thermal tolerance [25, 26], and enriching the cholesterol content of bovine cardiac microsomes improves the conformational stability of proteins and increases their resistance to inactivation at elevated temperature [27].

While a “high temperature” diet is available in Tasmania and other parts of the world (OPTILINE HT, Skretting), no peer-reviewed literature is available on its composition or efficacy, and this diet is not currently approved for use in Canada [28]. Further, as the Atlantic salmon industry is intending to produce 33,000 metric tonnes of all female triploid fish in Newfoundland by 2024 [29], producers must be proactive if they are to avoid events similar to that which occurred in 2019 [6]. This is particularly true, as while the data are equivocal, a number of studies suggest that triploid salmonids are less tolerant of high temperatures [30–34]. Finally, while farming all female triploid Atlantic salmon offers a highly effective strategy to mitigate the risk of genetic introgression by escapees [35], it is paramount that the industry develops strategies and methodologies to ensure that triploids are able to thrive at higher temperatures.

Therefore, the research conducted was both novel and practical in its design and provides information that should benefit the Atlantic salmon aquaculture industry in Canada and elsewhere. Increased ocean temperatures can lead to sublethal effects that result in loss of production and cause large-scale mortalities [6, 8], and this study provides the industry with vital information regarding the farming of female triploid Atlantic salmon at elevated temperatures.

## 2. Materials and Methods

This study was approved by the Animal Care Committee of Memorial University of Newfoundland and Labrador (protocol ^#^20-02-KG), and salmon husbandry and experimental procedures were performed in accordance with the Canadian Council on Animal Care Guidelines on the “Care and Use of Fish in Research, Teaching and Testing” [36].

### 2.1. Experimental Animals

PIT- (Passive Integrated Transponder-) tagged conventional female triploid Atlantic salmon of St. John River origin from AquaBounty Canada (PE, Canada) were shipped to the Laboratory for Atlantic Salmon and Climate Change Research (LASCCR; Ocean Sciences Centre, Memorial University) where they underwent smoltification. These fish were produced from a single reversed-sex neomale (i.e., functionally masculinized genetic female) that had been crossed with 24 females, producing all female offspring. Fertilized eggs were pooled and then shocked using hydrostatic pressure to induce triploidy [37]. Ploidy status of the fish was verified by AquaBounty using flow cytometry [38, 39] before transfer. Based on a subset of 200 eyed eggs, it was estimated that pressure shocking was ≥ 98% effective in inducing triploidy.

The fish were initially distributed among eight 2.2 m^3^ tanks at 16.5 kg m^−3^ in a flow-through seawater system with temperature set to 10°C for the first week before being slowly increased (i.e., at +0.3°C day^−1^) to 12°C over the course of another week. Tanks were maintained on a 14 h light : 10 h dark photoperiod with flow rates set to 15 L min^−1^. Oxygen and temperature levels were measured at least once daily in all tanks (YSI, ProODO, Yellow Springs, OH, USA), and 4 tanks were continuously tracked using a YSI 5500D Multi DO Optical Monitoring and Control System. Water oxygen levels were maintained at ≥ 100% air saturation, and the salmon were initially fed a commercial diet (EWOS Dynamic S, 5.0 mm; minimum 46% crude protein, 27% crude fat; EWOS Canada Ltd., Surrey, BC, Canada) to satiation twice daily by hand. Once the fish reached 12°C, they were redistributed amongst nine 2.2 m^3^ tanks with 50 fish tank^−1^ (initial stocking density ~9 kg m^−3^). Only fish weighing between 300 and 500 g were included in the study to limit the effect(s) of size variation. Salmon were then given another 18 days to acclimate before the initial assessment was performed and the trial started.

### 2.2. Overall Experimental Design and Fish Sampling

**Figure 1:**
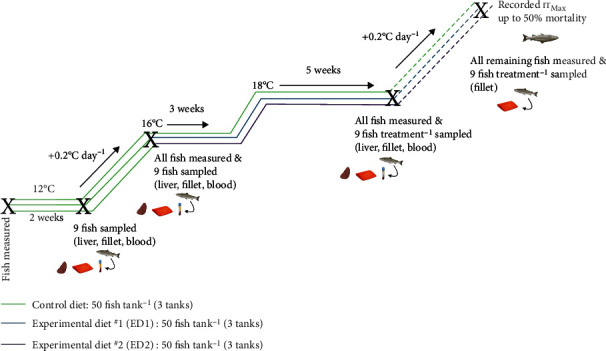
Overview of the protocol used to assess whether supplemental dietary cholesterol affected the growth performance, physiology and survival of female triploid Atlantic salmon when exposed to an increasing temperature regimen. ‘X's mark sampling time points. Created using https://biorender.com/.

An overview of the experimental design is shown in Figure 1. The initial weights and fork lengths of the fish were recorded after brief anesthesia (0.2 g L^−1^ AquaLife TMS; Syndel Laboratories Ltd., Nanaimo, BC, Canada) at 12°C. The fish were then allowed 2 days of recovery before their commercial diet was switched to the trial's control diet. All diets in this study were formulated to meet or exceed the nutritional requirements for Atlantic salmon [40] and approximate commercial salmon aquafeeds using practical ingredients (Table 1). The diets were extruded (5.0 mm pellet size) at the Chute Animal Nutrition Lab, Faculty of Agriculture, Dalhousie University (Truro, NS, Canada). Dry ingredients for both diets were mixed in an industrial 19 L Hobart mixer (Toronto, ON, Canada; Model A-200-T) for five minutes, then extruded into 5 mm pellets using a single screw KAHL extruder with mixing conditioner (Amandus Kahl GmbH & Co. KG, Reinbek, Germany; Type OEE8). The extruded pellets were dried in a shelf oven for eight hours at 60°C and sieved to remove fine particles. Pellets were then vacuum coated with dietary oils (Dinnissen Process Technology, Sevenum, Netherlands) and shipped to Memorial University of Newfoundland and Labrador. In addition to the control diet, 2 experimental diets containing supplemental cholesterol (+1.30% and 1.76% dietary cholesterol, respectively) were also manufactured. The proximate, lipid class, and fatty acid composition of all 3 diets can be found in Table 2. The amount of supplemental dietary cholesterol included in each experimental diet was selected in an attempt to match the 1.37 and 1.83% total cholesterol values reported in a study on a closely related species (i.e., rainbow trout; [23]). All diets contained approximately equal levels of protein (51%), lipid (20%), carbohydrate (12%), digestible energy (21 MJ kg^−1^), phosphorus (1.6%), eicosapentaenoic acid (EPA) and docosahexaenoic acid (DHA) (2% total of the diet), and astaxanthin (80 mg kg^−1^), with all essential amino acids supplied at a minimum of 150% of their known dietary requirement for Atlantic salmon [40]. Proximate composition and gross energy density of the test diets were measured following the procedures described in Tibbetts et al. [41], and their lipid classes and fatty acid profiles were determined following Wei et al. [42].

The salmon were hand fed the control diet to apparent satiation twice daily (at ~9 : 00 and 15 : 00) for 16 days at 12°C, with feeding stopped when a few pellets accumulated on the bottom of each tank. Then, the first sampling was performed. One fish was randomly netted from each tank (*n* = 9 in total), euthanized (0.4 g L^−1^ TMS), measured for weight and fork length, and sampled. Blood (1 mL) was collected within 2-3 min from each fish via the caudal vein using 1 mL syringes with 23 gauge 1^″^ needles and placed in heparinized (1000 units mL^−1^) tubes on ice. The blood was then centrifuged at 1100 × *g* for 1 min at room temperature; then 3 aliquots (100 *μ*L each) of plasma were collected before being flash-frozen in liquid nitrogen. The remaining blood was drained from the fish (via multiple collections using a 3 mL syringe and needle), the viscera was removed, and the liver was weighed. Next, duplicate pieces of liver from the most distal portion of the posterior lobe were sampled using standard aseptic techniques and quickly flash-frozen. The liver samples were stored at -80°C until one set of samples could be shipped to the Center for Aquaculture Technologies Canada (CATC; Souris, PE, Canada) for processing. The gonads were also examined to confirm that the fish were sterile females. The right side of each fish was then filleted, the bones were removed, and the skin-on fillet was placed on plain white paper inside a white Styrofoam box with a 100 W halogen light bulb hanging 87.5 cm directly above [43]. The fillets were then scored using the DSM SalmoFan™ colour chart [44], the industry-recognized standard for the visual assessment of the degree of pigmentation in salmon flesh. Two trained technical staff independently scored each fillet to determine its level of colouration, with separate scores assigned to describe the colouration along the lateral line and at the peripheral edges of the fillet. An average of these two values was used as the pigmentation score for each fillet. The fillets were then weighed, and samples of fillet muscle were collected and stored for further analyses conducted through the Mitigating the Impact of Climate-Related Challenges on Salmon Aquaculture (MICCSA) project. These data will be the basis of a separate paper.

The following day, the remaining fish were exposed to an incremental temperature increase from 12°C, where temperature was raised by 0.2°C day^−1^ to mimic conditions that these salmon may experience in sea cages during the summer in Newfoundland [45, 46] or northern Europe [47, 48]. Once the tanks reached 16°C, another sampling was performed following the same procedure as described above (*n* = 9; 1 fish sampled tank^−1^) with fish taken off feed 24 h prior to sampling. All remaining fish were also anesthetized (in 0.2 g L^−1^ TMS) and weighed and measured for fork length. A total of 25 fish were removed from the experiment at this point, as these salmon had developed ulcers (~3 cm × 3 cm on average) on their right side. Samples of these fish were taken and analyzed by the Microbial Pathogenesis and Vaccinology Laboratory (Memorial University), but the results were inconclusive (data not shown). The next day, 3 tanks were switched onto experimental diet ^#^1 (ED1; +1.30% cholesterol) and another 3 tanks were provided with experimental diet ^#^2 (ED2; +1.76% cholesterol). The remaining 3 tanks were kept on the control diet. The diets were switched at this stage of the experiment to reflect when salmon farmers would need to consider using a functional feed as temperatures warm. Temperature was maintained at 16°C for another 3 weeks before it was gradually increased (+0.2°C day^−1^) to 18°C, where temperature was again held for 5 weeks. No fish developed ulcers during the remainder of the experiment.

After exposure to elevated temperatures (i.e., ≥ 16°C) for a total of 65 days, another subset of fish was sampled after being fasted for 24 h. Nine fish per dietary treatment (3 fish tank^−1^) were sampled in the same manner as previously described at 12 and 16°C, with the addition that the viscera was weighed. Only fish that had gained weight were sampled, with 24 out of 27 fish having gained ≥ 10% of their weight since their assessment at 16°C and the remaining 3 sampled fish having gained between 5 and 10%. This was done to ensure that these fish were actively consuming the diets provided. Additional fish were sampled at this time point for separate analyses involved in the MICCSA project that will also not be described here. However, morphometric data collected from these fish are included in the current study.

Once all sampling was completed, temperature was again raised by 0.2°C day^−1^ in all tanks until 50% of the fish in each treatment reached their incremental thermal maximum (IT_Max_). When each fish lost equilibrium/succumbed, their weight, fork length, liver weight, viscera weight, ventricle weight, and state of sexual maturity (or lack thereof) were recorded, in addition to the time and temperature at which they were removed from the experiment. After the first dietary treatment reached this endpoint, 9 fish per dietary treatment (3 fish tank^−1^) were euthanized (0.4 g L^−1^ TMS), measured for weight and fork length, and sampled. Viscera, liver, and ventricle weight were also measured. Fillet weight and colouration were assessed as previously described. In addition, after 50% of the fish within each dietary treatment reached their IT_Max_, the remaining fish were euthanized (0.4 g L^−1^ TMS) before weight and fork length were recorded.

### 2.3. Growth Performance

Weight gain was assessed at 16°C, at the end of 5 weeks spent at 18°C, and when fish reached their IT_Max_. The thermal growth coefficient (TGC) was used to assess growth rate using the following equation [49, 50]:

(1) $$\begin{array}{c} \text{TGC}=\left(\frac{{W_{f}}^{1/3}-{W_{i}}^{1/3}}{\sum_{i=1}^{n}Ti}\right)\times 1000, \end{array}$$

where *W*_*f*_ and *W*_*i*_ are the final and initial fish body weights (in g), respectively, *n* is the number of days since *W*_*i*_, and *T*_*i*_ is the mean daily water temperature (in °C).

Specific growth rate (SGR) was also calculated using

(2) $$\begin{array}{c} \text{SGR}=\left(\frac{\ln\left(W_{f}\right)-\ln\left(W_{i}\right)}{n}\right)\times 100. \end{array}$$

Fulton's condition factor (*K*) was calculated as

(3) $$\begin{array}{c} K=\frac{\text{Fish weight }\left(\text{g}\right)\;}{{\left(\text{Fish fork length }\left[\text{cm}\right]\right)}^{3}}\times 100. \end{array}$$

Feed intake was measured daily by dividing the feed provided to each tank by the number of fish in the tank. Average feed intake was also calculated on a percent body weight basis at the assessment points. Liver, viscera, ventricle, and fillet weights were used to calculate each fish's hepatosomatic index (HSI), viscerosomatic index (VSI), relative ventricular mass (RVM), and fillet yield, respectively, using the following equations:

(4) $$\begin{array}{c} \text{HSI}=\left(\frac{\text{Liver weight }\left(\text{g}\right)\;}{\text{Fish weight }\left(\text{g}\right)}\right)\times 100,\\ \text{VSI}=\left(\frac{\text{Viscera weight }\left(\text{g}\right)\;}{\text{Fish weight }\left(\text{g}\right)}\right)\times 100,\\ \text{RVM}=\left(\frac{\text{Ventricle weight }\left(\text{g}\right)\;}{\text{Fish weight }\left(\text{g}\right)}\right)\times 100,\\ \text{Fillet yield}=\left(\frac{\text{Fillet weight }\left(\text{g}\right)*2\;}{\text{Fish weight }\left(\text{g}\right)}\right)\times 100. \end{array}$$

### 2.4. Basal Cortisol Levels

Plasma cortisol was measured at 650 nm using a commercial enzyme-linked immunosorbent assay (ELISA) kit (Neogen, Lexington, KY, USA) and a Molecular Devices (San Jose, CA, USA) SpectraMax® M5 microplate reader, following the manufacturers' instructions. Samples were analyzed in duplicate at dilutions of 1 : 50, with a duplicate 8-point standard curve included on each 96-well plate. A 4-parameter logistic fit of the standard curve gave *r*^2^ values of ≥0.994 for each plate. Samples with coefficients of variance > 15% and with a >10 ng mL^−1^ difference in cortisol level between replicates were repeated. It is acknowledged that the commercial antibody used in this ELISA can cross-react with other steroids (i.e., cortisone, 11-deoxycortisol, and corticosterone), and thus, absolute cortisol concentrations cannot be inferred [51].

### 2.5. Liver RNA Extraction, Purification, and cDNA Synthesis

Liver samples (*n* = 45) were shipped on dry ice to CATC for transcript expression analyses. Isolation of RNA from the liver was completed as per Xue et al. [52], with the exception of DNase treatment and purification, which was completed with a RNA Clean & Concentrator Kit (Zymo Research, Irvine, CA, USA) as per the manufacturer's instructions. The purity of RNA was determined by spectrophotometry with 260/280 and 260/230 values of ≥2.0 and 1.9, respectively, as minimum thresholds for inclusion. RNA integrity was confirmed using 1.0% agarose gel electrophoresis. The RNA samples were standardized to 100 ng/*μ*L, and synthesis of cDNA was completed from 1 *μ*g of RNA using the High Capacity cDNA Reverse Transcription Kit (Applied Biosystems, Foster City, CA. USA) as per the manufacturer's instructions.

### 2.6. RT-qPCR Study and Analysis

Seven genes of interest were chosen based on past studies that have shown them to be responsive to chronic heat stress in Atlantic salmon liver [53, 54]. Several heat shock proteins [i.e., *hsp70*, *hsp90aa1*, *hsp90ab1*, and *serpinh1* (alias *hsp47*)] were selected alongside transcripts associated with oxidative stress (i.e., *cirbp*, *ndufa1*, and *ucp2*). All qPCR primers, including the 5 normalizers (see below), were taken from previous studies [53–58]. An equimolar pool of cDNA from all samples was used as a calibrator sample between plates and as a source material for standard curve generation. Standard curves for all 12 transcripts were generated using four to six serial dilutions (1 : 3 or 1 : 5) from 5-10 ng of input total RNA. Linearity (*r*^2^) ≥ 0.980 and efficiency between 90 and 109% (Table 3) met quality standards [59]. “No reverse transcriptase” controls, using pools of 22-23 samples, did not reveal signs of gDNA contamination. Melt curves indicated sharp, single peaks with no evidence of primer dimers.

A 1 : 20 dilution of stock cDNA was used for qPCR analyses. Each reaction was 10 *μ*L and included 5 *μ*L of SsoAdvance Universal SYBR Green Supermix (BioRad, Saint-Laurent, QC, Canada), 400 nM of each primer, and 3 *μ*L of template (7.5 ng input RNA). Thermal cycling on the 7500 FAST qPCR System (Applied Biosystems) included an initial activation and denaturation step of 95°C for 2 min followed by 40 cycles of 95°C for 5 sec and 60°C for 30 sec. The only exceptions to these parameters were for the following: (1) *cirbp*, for which we used 250 nM of each primer and a 62°C annealing step, and (2) *hsp90aa1*, for which we used 500 nM of each primer and a 45 sec annealing step. Melt curve analysis included 95°C for 15 sec, 60°C for 1 min, and a 0.5°C/sec ramp to 95°C. Each 384-well plate included triplicate samples, calibrators, and nontemplate controls (NTCs) for each gene present.

Raw cycle threshold (C_T_) data were imported into qbase+ (Biogazelle, Gent, Belgium) [60], where technical replicates outside of ±0.5 C_T_ from two close values were removed. While 5 normalizer genes were initially assessed (i.e., *ef1a*, *rpl32*, *eif3d*, *pabpc1*, and *polr2*), *ef1a* and *rpl32* were ultimately selected as the study normalizers. This selection was based on low C_T_ variation among temperature/dietary groups (i.e., <1 C_T_ difference in averages between groups) and geNorm recommendations (mean geNorm *M* value and coefficient of variation of 0.342 and 0.119, respectively) [61]. Calibrated normalized relative quantities (CNRQs) [61] were calculated using C_T_ values of calibrators across plates and amplification efficiencies for each primer pair (Table 3) in qbase+. CNRQs were then log_2_-transformed in Microsoft Excel.

### 2.7. Statistical Analyses

Data were first assessed via Shapiro-Wilk's normality tests and log_10_-transformed if necessary to meet testing assumptions. Levene's tests to measure homoscedasticity were also performed. To confirm the absence of tank effects, replicate tank means were first compared, with tank as a fixed factor, after which the data from replicate tanks were pooled for further analyses. One-way ANOVAs followed by Tukey's HSD post hoc tests were used to examine differences (*p* < 0.05) in parameters between (1) the dietary treatments at a particular temperature and (2) temperatures within just the control treatment. *T*-tests were used when comparisons were only made between two variables (e.g., assessments performed at 12°C vs. 16°C). Data are reported as means ± standard error (SE). All statistical procedures were carried out using R (v. 4.1.2) [62].

## 3. Results

### 3.1. Performance Metrics following an Incremental Temperature Increase to 18°C

**Figure 2:**
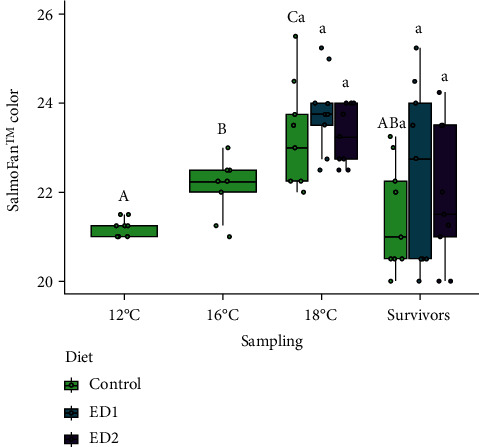
SalmoFan™ fillet colour scores over the course of the study (*n* = 9 per each sampling/dietary treatment). Upper case letters denote significant (*p* < 0.05) differences between samplings within the control treatment. Lower case letters indicate that no differences were detected among dietary treatments within a sampling. There was no difference in the colour scores in fish fed experimental diet ^#^1 (ED1) and ^#^2 (ED2) between samplings.

**Figure 3:**
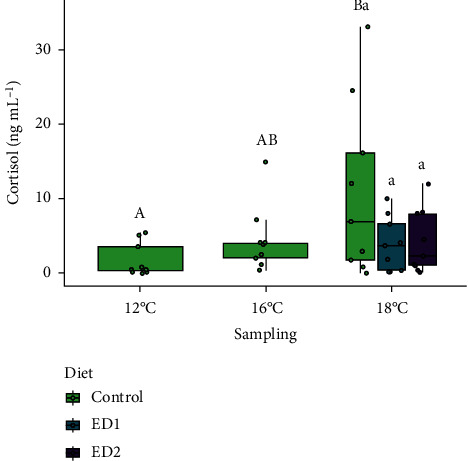
Basal (resting) plasma cortisol concentrations over the course of the study (*n* = 9 per each sampling/dietary treatment). Upper case letters denote significant (*p* < 0.05) differences between samplings within the control treatment. Lower case letters indicate that no differences were detected among dietary treatments at 18°C.

Performance metrics of the fish sampled at 12 and 16°C can be found in Table 4. The fish gained almost 100 g, on average, during the 37-day initial period where all fish were fed the control diet as temperature was gradually raised to 16°C from 12°C. This resulted in values of TGC and SGR over this period of 0.85 ± 0.03 [g^1/3^ (°C d)^−1^] and 0.41 ± 0.02 (% body weight day^−1^), respectively. Although Fulton's condition factor decreased slightly (*p* < 0.001) in salmon at 16°C as compared to those assessed at 12°C, there were no differences in feed intake, HSI, or fillet yield between the two assessment points; values for these parameters were approx. 0.75% body weight day^−1^, 1.26% and 51.2%, respectively. SalmoFan™ colouration values for the fillet increased by one point on average (*p* < 0.001) from 12 to 16°C, indicating that pigmentation of the fillet intensified as the fish grew (Figure 2). Basal cortisol levels (Figure 3) and expression of most heat stress biomarkers (i.e., *cirbp*, *hsp70*, *hsp90aa1*, *hsp90ab1*, and *ndufa1*) were unaffected by this 4°C increase. In contrast, *serpinh1* and *ucp2* were both downregulated at 16°C as compared to at 12°C.

**Figure 4:**
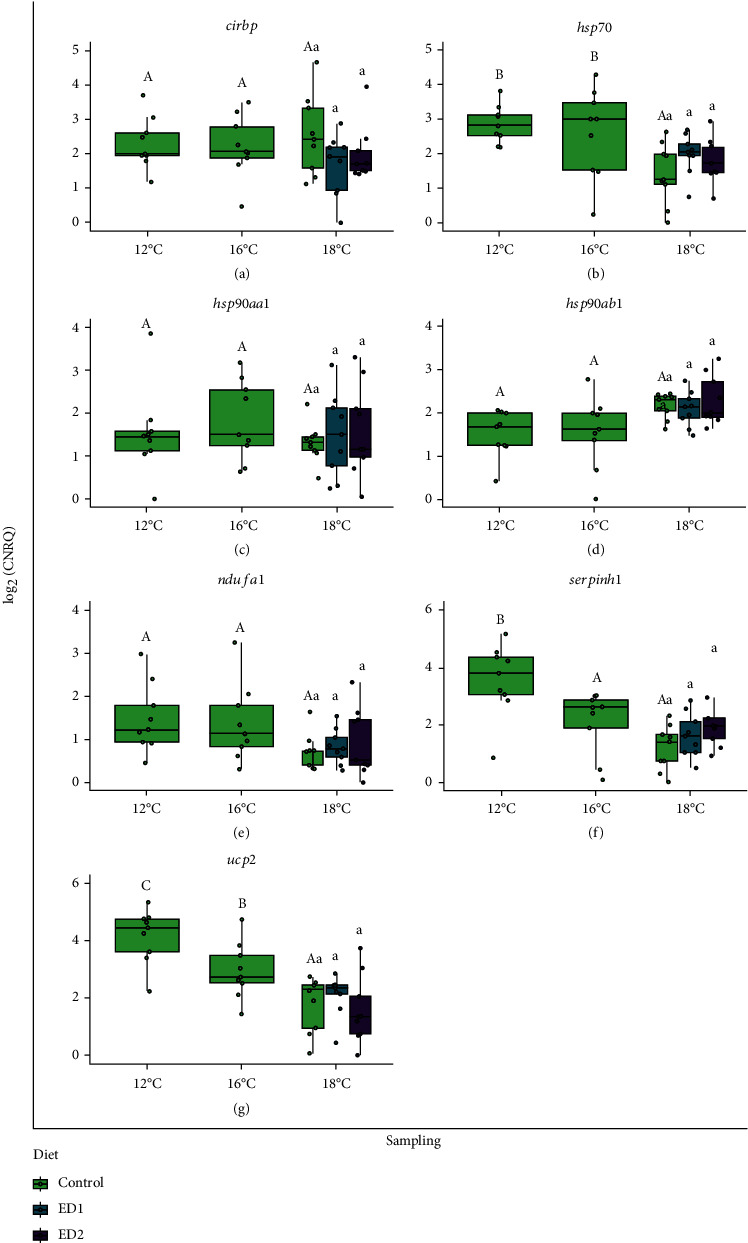
Expression levels of 7 transcripts with roles in responding to heat stress [*cirbp* (a), *hsp70* (b), *hsp90aa1* (c), *hsp90ab1* (d), *ndufa1* (e), *serpinh1* (f), and *ucp2* (g)] in the liver of female triploid Atlantic salmon sampled at 12, 16, or 18°C and provided different amounts of dietary cholesterol. Calibrated normalized relative quantities (CNRQs) were compared by one-way ANOVA (*p* < 0.05; *n* = 9 per sampling/diet). Upper case letters denote significant differences between samplings within the control treatment. Lower case letters are the same between diets as no significant differences were detected at 18°C. In all cases, the letter “A” signifies the lowest value within a comparison.

Fish sampled at 18°C in all dietary treatments, on average, gained weight (~50 g; *p* < 0.01) from when they were assessed at 16°C (Table 5). However, it is noteworthy that 41.3% of all fish lost weight since the 16°C assessment (-49 g on average), whereas the remaining 58.7% of the fish gained weight (+199 g on average). Feed intake decreased significantly (*p* < 0.001) compared to both previous assessments across all three of the dietary treatments (i.e., by 50.8% and 43.8% on average as compared to those measured at 12 and 16°C, respectively). Similarly, growth rate (i.e., as assessed by TGC and SGR) and condition factor declined by 78.0%, 74.0% and 11.2% (*p* < 0.001), respectively, in salmon reared at 18°C as compared to previous measurements (compare data in Tables 4 and 5). HSI was also lower (*p* < 0.05) at 18°C as compared to at 12 and 16°C in the control group. SalmoFan™ colour increased significantly (by 1.1 points on average, *p* < 0.05) in the control treatment as compared to when it was assessed at 16°C. No differences were found in fillet yield between any of the three sampling points (temperatures). However, basal plasma cortisol increased (*p* < 0.05) at 18°C compared to at 12°C (average values of 10.9 and 1.8 ng mL^−1^, respectively). The only transcripts found to be differentially expressed at 18°C were *ucp2* (significantly downregulated from 12 and 16°C), *hsp70* (significantly downregulated from 12 and 16°C), and *serpinh1* (significantly downregulated from 12°C). No differences in any parameter were identified between the three dietary treatments at 18°C (Tables 4 and 5; Figures 2–4).

### 3.2. Performance Metrics and Survival following an Incremental Temperature Increase until 50% Mortality

**Figure 5:**
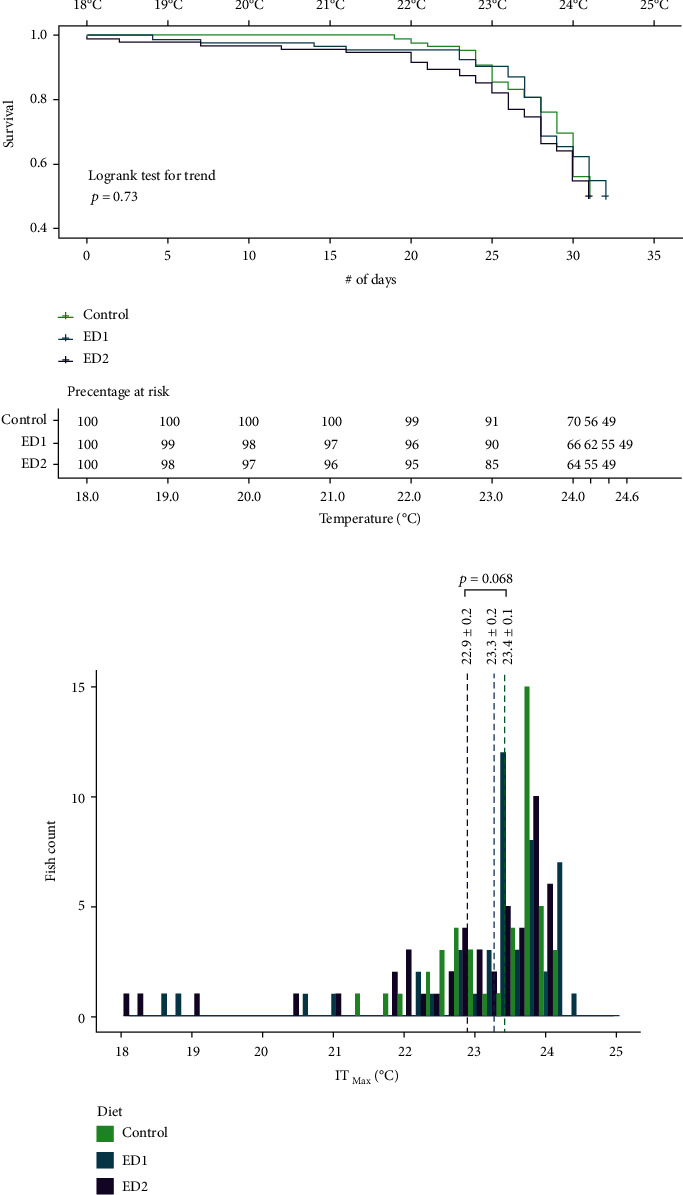
The incremental thermal maximum (IT_Max_ at 50% mortality in each dietary treatment) of female triploid Atlantic salmon exposed to a temperature increase of 0.2°C day^−1^ from 18°C after prolonged exposure to elevated temperatures (≥16°C) for 65 days. Fish were fed a control diet, or one of two diets supplemented with cholesterol (ED1 and ED2). (a) Kaplan-Meier survival curves are shown along with the results of the log-rank test to determine significance. (b) Histogram of the same dataset. The dashed lines indicating the average IT_Max_ value for the first 50% of fish that succumbed to high temperature for each dietary treatment.

Feed intake declined steeply after temperature increased above 18°C in all tanks, to a point where hardly any fish were feeding after 19°C (Supplemental Figure S1). A Kaplan-Meier curve with survival probabilities for each dietary group and a histogram of the IT_Max_ data are shown in Figure 5. Overall, there were very few fish that succumbed to the increasing temperature regimen before 22°C (< 5%). However, mortalities increased as the temperature rose further, and the average IT_Max_ at 50% mortality was 22.9 ± 0.2, 23.3 ± 0.2, and 23.4 ± 0.1°C in the ED2, ED1, and control groups, respectively (Figure 5(b), Table 6). Although a logrank test on survival indicated that the Kaplan-Meier curves were not different between the 3 groups (Figure 5(a); *p* = 0.73), the IT_Max_ values at 50% mortality were higher in control fish as compared to ED2 fish at a *p* value of 0.068. The study endpoints (i.e., 50% mortality within a particular treatment) were reached at 24.2, 24.4, and 24.0°C in the control, ED1, and ED2 groups, respectively.

Morphological and production metrics of the first 50% of fish within each dietary treatment that reached their IT_Max_ can be found in Table 6. Fish in all groups weighed significantly less (by ~29.9%; *p* < 0.001) than when they were assessed at 18°C and their condition factor scores were lower (by ~18.2%, *p* < 0.001), but they had higher HSI values (by ~8.6%, *p* < 0.05) (compare data in Tables 5 and 6). Interestingly, although no difference was detected within the ED2 dietary group, VSI scores were lower (*p* < 0.05) in both the control and ED1 treatments at 50% mortality (IT_Max_) compared to at 18°C.

Fish that survived to the endpoint of the experiment (i.e., after 50% of fish in each dietary treatment reached their IT_Max_), weighed more (*p* < 0.01), lost less weight (*p* < 0.01), were longer (*p* < 0.01), and had higher condition factor values (*p* < 0.05) as compared to fish within their respective dietary treatment that succumbed (compare Tables 6 and 7). However, these parameters were not different between the groups when survivors were compared (Table 7). SalmoFan™ colour scores for the control group were significantly less at this sampling point than when measured at 18°C. In contrast, no such decrease in SalmoFan™ colour score was observed in fish in the ED1 and ED2 groups (Figure 2).

## 4. Discussion

The objective of this research was to determine if supplementary dietary cholesterol could improve the upper thermal tolerance and growth performance of female triploid Atlantic salmon, a commercially important farmed fish in Newfoundland, Canada. Further, this study examined if adding cholesterol to salmon diets could preserve/protect fillet pigmentation in fish exposed to rising temperatures or alter the temperature-induced stress response. Supplemental dietary cholesterol did not improve survival or enhance growth at elevated temperatures or alter the hepatic expression of heat stress-associated transcripts, and may have negatively affected survival at the highest inclusion level (+1.76% cholesterol). However, adding cholesterol to the diet did limit fillet bleaching (i.e., loss of pigmentation) from occurring (at least in some salmon), as only the control treatment saw a significant decrease in SalmoFan™ scores after 50% of fish reached their IT_Max_, and potentially reduced basal cortisol (i.e., physiological stress) levels at elevated temperatures.

### 4.1. Growth Performance

Interestingly, a high proportion (i.e., > 40%) of salmon lost weight when reared at temperatures between 16 and 18°C. It is possible that this was directly related to triploidy or the particular stock used, and that they did not increase feed intake as temperature rose and feed intake fell noticeably at 19°C. For example, it has been previously reported that triploid Atlantic salmon exhibit reduced feed intake at elevated temperatures (i.e., 18°C) as compared to diploids [32, 33]. AquAdvantage Salmon (growth hormone transgenic female triploid Atlantic salmon produced by AquaBounty) have reduced values for TGC at temperatures above 16°C compared to 10.5 and 13.5°C [43]. Further, Ignatz et al. [63] showed that conventional male diploid Atlantic salmon sourced from AquaBounty Canada and exposed to a similar incremental thermal challenge, increased their feed intake until 22°C. However, this finding may have also been influenced by other factors. First, a small proportion of fish in this study developed dermal sores before the assessment at 16°C, so it is possible that some fish were combatting an unknown infection despite the absence of clinical signs (i.e., did not develop dermal sores like other fish). Second, while genetic variation in this study was low (i.e., all fish were full- or half-siblings), there is still potential that female parentage influenced growth performance at elevated temperatures. Finally, feed intake in the current experiment regardless of rearing temperature was lower than anticipated. At the start of the experiment, when fish were abruptly switched from a commercial feed to the control diet, feed intake dropped by ~39.3% over the next 2 weeks before increasing again (Supplemental Figure S1). A more gradual transition between the commercial feed and the test control diet may have better maintained feed intake during the early portion of the study [64].

### 4.2. Fillet Pigmentation

It is paramount that salmon aquaculture producers maximize fillet pigmentation/astaxanthin levels. High astaxanthin retention is associated with higher total antioxidant capacity in juvenile rainbow trout [65]; a potentially useful trait in fish exposed to elevated temperatures. In addition, darker fillet pigmentation is more attractive to consumers [66]. Yet astaxanthin is both an expensive [20] and a restricted additive (i.e., regulations limit dietary carotenoid inclusion at ≤ 80 mg kg^−1^ feed) [67]. Several studies have reported a decrease in fillet astaxanthin concentration/pigmentation in adult Atlantic salmon during the summer months (i.e., at high water temperatures), which is likely associated with increased oxidative stress and the mobilization of astaxanthin to maintain redox homeostasis [8, 12, 13]. A significant decrease in SalmoFan™ colouration at 50% mortality as compared to at 18°C was only observed in the control group in the current study, and this suggests that supplemental cholesterol reduced astaxanthin mobilization or increased absorption/retention [21, 22].

While SalmoFan™ scores increased between 16 and 18°C, it is noteworthy that values only increased by ~1 point, the same amount that they increased during the short-term transition from 12 to 16°C (20 days). These data suggest that astaxanthin retention was difficult for triploid salmon at temperatures ≥ 16°C, and the results of Ignatz et al. [43] support this hypothesis. SalmoFan™ scores were lower in 1.5 kg AquAdvantage Salmon reared at 16.5°C compared to at 13.5°C [43]. Single-nucleotide polymorphisms (SNPs) for dual oxidase 2 (*duox2*) and dual oxidase maturation factor 1 (*duoxa1*) have been identified in Atlantic salmon hindgut samples via RNA-sequencing (RNA-seq) that are associated with high (i.e., SalmoFan™ scores ≥ 25 following summer rearing) and low fillet colouration (i.e., SalmoFan™ scores ≤ 24, respectively [68]. It would be valuable in future studies to examine if these types of markers can be validated in salmon exposed to IT_Max_ protocols using either RNA-seq-based identification of trait-associated SNPs or SNP genotyping using gDNA.

### 4.3. Stress Indices

Overall, the cortisol and hepatic transcript expression data suggest that female AquaBounty triploid salmon that were still feeding were not experiencing stress at temperatures up to 18°C. However, at 18°C, basal cortisol levels increased slightly in the control treatment compared to measurements at 12°C (Figure 3). While no significant differences were detected between dietary treatments at 18°C, average cortisol levels were 64.2% and 61.5% lower in the ED1 (*p* = 0.14) and ED2 (*p* = 0.16) groups, respectively, as compared to the controls. Therefore, supplemental cholesterol may potentially lower resting cortisol concentrations. The current results agree with past findings on ~800 g AquAdvantage Salmon, where no differences in basal cortisol levels were detected between fish reared at 10.5, 13.5, or 16.5°C [69]. While it could be hypothesized that supplemental dietary cholesterol might increase basal cortisol concentrations, as cholesterol is a precursor in cortisol synthesis [70], this was not observed in the current study. It is possible that the additional cholesterol assisted in maintaining cell membrane rigidity [15] and ultimately reduced cellular and overall stress. Differences in cortisol regulation could have also altered gluconeogenesis in the liver and modulated energy (e.g., lipid) stores within the fish [71]. However, these hypotheses would need to be tested.

In contrast, the transcript expression of heat stress biomarkers measured in the liver does not align well (with the exception of *ucp2*) with past investigations of chronic/prolonged heat stress in Atlantic salmon [53, 54, 72]. It was anticipated that the expression of heat shock proteins (i.e., *hsp70*, *hsp90aa1*, *hsp90ab1*, and *serpinh1*) would be upregulated and that expression of transcripts associated with oxidative stress (i.e., *cirbp*, *ndufa1*, and *ucp2*) would be downregulated at 16 and/or 18°C as compared to at 12°C [53, 54, 72]. However, in this study, we report that *hsp70* and *serpinh1* were downregulated at 16 and/or 18°C compared to at 12°C and that temperature had no effect on the expression of *cirbp*, *hsp90aa1*, *hsp90ab1*, and *ndufa1* (Figure 4). While it is not surprising that some heat shock proteins were not yet upregulated at 16°C, an incremental temperature increase (+1°C week^−1^) up to 18°C from 12°C previously increased the expression of *hsp70*, *hsp90aa1* and *serpinh1* in diploid postsmolt Atlantic salmon [72]. The fact that fish in the current study spent 65 days at temperatures ≥ 16°C before sampling at 18°C could potentially explain the differences observed. Prolonged exposure to elevated temperatures could lead to acclimatory responses, thus limiting the requirement for heat shock proteins to prevent protein damage or denaturation. Although, 800 g AquAdvantage Salmon reared in freshwater at 16.5°C for several months still exhibited higher expression of *hsp90ab1* and *serpinh1* and lower expression of *cirbp* compared to salmon reared at 10.5°C [54]. The differences in time spent at elevated temperatures, genetic background, water salinity, ploidy, and/or transgenesis make it difficult to directly compare with past results [54, 72].

It is also possible that this discrepancy was due to the fact that the fish were potentially exposed to an unknown pathogen from 12 to 16°C. However, this hypothesis is quite speculative. Eslamloo et al. [73] showed that *hsp70*, *hsp90aa1*, and *cirbp* transcript expressions were upregulated, while that of *serpinh1* and *ucp2* was downregulated, in the skin of ~200 g (8°C acclimated) diploid Atlantic salmon exposed to *Moritella viscosa*, whereas liver *hsp70*, *hsp90aa1* and *ucp2* transcriptions were upregulated in ~500 g male diploid Atlantic salmon reared at 12 or 20°C and injected with a commercial multivalent vaccine (i.e., Forte Micro; containing formalin-inactivated cultures of several bacterial pathogens) (Ignatz et al., in prep.).

Collectively, this body of research/data indicates that more research needs to be conducted to elucidate the impact that chronic exposure to elevated temperatures has on the hepatic transcript expression of female triploid Atlantic salmon. Such research could also help to identify potential paralogue-specific differences in transcript expression.

### 4.4. Survival at Elevated Temperatures

It has been suggested that triploid salmonids have reduced survivorship at elevated temperatures compared to diploids [31, 74], although this phenomenon is not observed consistently [75–77]. This is the first study to expose triploid Atlantic salmon to an incremental temperature increase (i.e., an IT_Max_ test) that mimics the natural conditions that these fish are expected to encounter in the North Atlantic as ocean temperatures rise. In the current study, our triploid all-female salmon did not experience large numbers of mortalities until after 22°C, which is similar to what has been reported for diploid mixed-sex Atlantic salmon exposed to comparable gradual increases in temperature [78, 79]. However, it must be noted that (1) these fish are from a commercial farm that has held salmon for 25 years (~8 generations) in land-based systems where the lowest temperature they experience is 6°C; (2) that long-term rearing conditions can have a significant effect on a fish's response to environmental conditions (e.g., [80, 81]); and (3) that diploid male salmon from AquaBounty have an even higher IT_Max_ (by >1°C) at which 50% mortality occurred than the fish used in the current study [63]. Thus, the high IT_Max_ values reported for all-female triploid salmon may be specific to AquaBounty's stock. It is also possible that, in comparison to previous IT_Max_ tests on diploid Atlantic salmon where fish did not spend an extended period at ≥ 16°C [63, 78, 79], the IT_Max_ of the current triploids may be underestimated. Nonetheless, the most important finding here is that triploid all-female salmon can be developed that are able to withstand the highest summer temperatures that are reported in Atlantic Canada and in Europe [6, 46–48]. Advances in genotyping methods could lead to the selection of Atlantic salmon (stocks) with even higher values for upper thermal tolerance, as this trait has been shown to be heritable in other salmonids [82–84]. Indeed, analyses of AquaBounty's genetics in relation to IT_Max_ are ongoing.

## 5. Conclusions and Perspectives

The results of this study are particularly relevant to the production of female triploid Atlantic salmon. As the triploid salmon used in this study did not experience high mortalities until after 22°C, this suggests that their production is potentially suitable for the North Atlantic. However, further study is required to verify if this is true across all farmed triploid populations, or specific to those reared in the land-based facilities at AquaBounty Canada.

Additional research is also recommended into the use of dietary manipulation to enhance the production of farmed all-female triploid Atlantic salmon. While the current results suggest that supplemental cholesterol may not be beneficial to Atlantic salmon reared at elevated temperatures, other ingredients/additives (e.g., prebiotics, vitamins C and E, and selenium) show promise for improving thermal tolerance in and/or offsetting the negative impacts of heat stress on, other fish species [85–88]. It also remains to be determined/reported what effect additional dietary cholesterol has on other important characteristics (e.g., the innate antibacterial and antiviral immune responses or tissue lipid composition) in the salmon used in the current study (although these data are currently being analyzed; Ignatz et al., in prep.). Therefore, it is still possible that increased dietary cholesterol may have benefited these salmon in other ways.

Finally, it may also be worthwhile to test lower inclusion levels of supplemental cholesterol. In this study, we attempted to match the 1.37 and 1.83% total dietary cholesterol values reported in Deng et al. [23], where at the lower inclusion level, supplemental cholesterol enhanced antioxidant activity and survival of rainbow trout following injection with *A*. *hydrophila*. However, total sterols (Table 2) in ED1 and ED2 were both higher, at 2.13 and 2.54%, respectively. Dietary noncholesterol sterols (i.e., phytosterols) are included in the total sterol amounts, and possible positive effects of supplemental cholesterol may have been negated by them. However, in this regard, the data are again ambiguous. Whereas Deng et al. [23] suggest that positive effects of supplemental cholesterol may not be seen at such high inclusion levels, the addition of 2.0-2.69% cholesterol improved astaxanthin absorption in preadult Atlantic salmon reared at 10 and 12°C without any mention of negative effects [21, 22]. Overall, the results of the current study provide novel insight into the roles that both rearing temperature and dietary cholesterol may have on the production of female triploid Atlantic salmon. However, further research will help optimize strategies to improve performance in these farmed populations.

## Figures and Tables

**Table 1:** Formulation of the test diets used to evaluate the effect(s) of supplemental dietary cholesterol on female triploid Atlantic salmon exposed to elevated temperatures.

Ingredient^1^ (% of diet)	Control diet	Experimental diet ^#^1 (ED1)	Experimental diet ^#^2 (ED2)
Fish meal (69% CP^2^)	18.00	18.00	18.00
Soybean protein concentrate (71% CP)	17.00	17.00	17.00
Soybean meal (48% CP)	12.00	12.00	12.00
Poultry by-product meal (71% CP)	12.00	12.00	12.00
Blood meal (96% CP)	6.72	6.72	6.72
Fish oil	9.00	8.29	8.04
Wheat flour	8.48	8.60	8.64
Poultry fat	4.50	4.15	4.02
Canola oil	4.50	4.15	4.02
Calcium phosphate (monobasic)	4.67	4.67	4.67
Vitamin/mineral premix^3^	0.40	0.40	0.40
Vitamin B4 (choline chloride)	0.40	0.40	0.40
Vitamin C (ascorbic acid; “stay-C 35”)	0.03	0.03	0.03
Vitamin E (*α*-tocopherol)	0.03	0.03	0.03
L-lysine	1.68	1.68	1.68
DL-methionine	0.50	0.50	0.50
L-tryptophan	0.01	0.01	0.01
Carophyll pink (10% astaxanthin)	0.08	0.08	0.08
Cholesterol^4^	0.00	1.30	1.76

^1^All ingredients were supplied by Corey Nutrition (Fredericton, NB, Canada), with the exception of the supplemental cholesterol. ^2^Crude protein (CP; *N* × 6.25). ^3^Seawater salmonid mixture. ^4^Sigma-Aldrich (Oakville, ON, Canada), CAT^#^ C8503.

**Table 2:** Proximate, lipid class, and fatty acid composition of the experimental diets fed to the female triploid Atlantic salmon.

	Control diet	Experimental diet ^#^1	Experimental diet ^#^2
	No added cholesterol	1.30% added cholesterol	1.76% added cholesterol
*Proximate composition^1^*		
Moisture (%)	5.6	6.3	6.1
Ash (%)	10.1	10.4	10.6
Crude protein (%)	51.4	51.7	51.2
Crude lipid (%)	20.5	19.1	19.3
Carbohydrate^2^ (%)	12.4	12.5	12.8
Gross energy (MJ kg^−1^)	21.8	21.5	21.4
*Lipid class composition^3^*		
Triacylglycerol	115.0	122.1	128.3
Sterol	3.4	18.8	24.5
Phospholipid	23.4	4.9	15.9
Free fatty acid	6.8	11.8	10.3
Acetone mobile polar lipid	21.4	8.7	6.4
Total lipid	171.9	168.8	186.3
*Fatty acid composition^3^*		
14 : 0	6.2	5.4	6.0
16 : 0	22.8	21.5	23.9
16 : 1*ω*7	9.6	8.6	9.5
18 : 0	5.3	5.1	5.6
18 : 1*ω*9	37.9	39.1	38.9
18 : 1*ω*7	3.8	3.6	3.9
18 : 2*ω*6 (LNA)	18.3	18.7	20.4
18 : 3*ω*6	0.3	0.3	0.3
18 : 3*ω*3 (ALA)	4.0	4.1	4.1
18 : 4*ω*3	1.3	1.1	1.3
20 : 1*ω*9	2.4	2.5	3.0
20 : 2*ω*6	0.2	0.1	0.1
20 : 3*ω*6	0.2	0.1	0.2
20 : 4*ω*6 (ARA)	0.9	0.8	1.1
20 : 3*ω*3	0.1	0.1	0.1
20 : 4*ω*3	0.6	0.5	0.6
20 : 5*ω*3 (EPA)	8.7	7.4	8.3
22 : 5*ω*3	1.3	1.1	1.3
22 : 6*ω*3 (DHA)	5.4	4.8	5.5
*Σ* SFA^4^	37.2	34.6	38.4
*Σ* MUFA^5^	58.4	58.6	61.0
*Σ* PUFA^6^	45.0	42.6	46.8
*Σ ω*3	21.8	19.5	21.5
*Σ ω*6	20.3	20.6	22.6
*ω*6/*ω*3	0.9	1.1	1.1
DHA/EPA	0.6	0.7	0.7
EPA/ARA	9.7	9.3	7.6

^1^Data presented on an as-fed basis. ^2^Estimated as (100% − [Moisture + Ash + Crude protein + Crude lipid]). ^3^Data presented on a mg lipid or FAME g^−1^ wet weight basis. ^4^Saturated fatty acid. ^5^Monounsaturated fatty acid. ^6^Polyunsaturated fatty acid.

**Table 3:** qPCR primers used for assessing heat stress responses in the liver of female triploid Atlantic salmon.

Gene name (GenBank accession number)	Nucleotide sequence (5′-3′)	Amplification efficiency (%)	*r* ^2^	Amplicon size (bp)	Source
*Cold-inducible RNA-binding* *protein* (*cirbp*) (BT059171)	F: TTGAGTACACAGCGGTGAATT	93.4	0.980	132	Beemelmanns et al. [72]
	R: ACCAATCTGATGCTATGACGAGA				
*Heat shock protein 70* (*hsp70*) (BT045715)	F: AGTGATCAACGACTCGACACG	91.7	0.988	151	Beemelmanns et al. [72]
	R: CACTGCATTGGTTATAGTCTTG				
*Heat shock protein 90-alpha* (*hsp90aa1*) (KC150878)	F: CGAGGACATGAAGAAGAGGCAT	104.5	0.987	104	Beemelmanns et al. [72]
	R: ACACTGTCACCTTCTCCACTTT				
*Heat shock protein 90 alpha* *family class B member 1* (*hsp90ab1*) (NM_001123532)	F: AGCCTCACGTTTTTCCAATCG	91.6	0.994	150	Ignatz et al. [54]
	R: TGCGTTGCCCACCATTAACT				
*NADH dehydrogenase 1* *alpha subcomplex subunit* *1* (*ndufa1*) (BT046880)	F: TGATGGAGAGAGACAGACGAGT	96.7	0.988	89	Beemelmanns et al. [53]
	R: AGGTGAGATCTGGGATTAGTGGA				
*Serpin H1* (*serpinh1*) (XM_014214963)	F: GACCATTCAAAAATCAACCTCA	94.0	0.986	129	Beemelmanns et al. [53]
	R: CATGGCTCCATCAGCATTCT				
*Mitochondrial uncoupling* *protein 2* (*ucp2*) (XM_014196911)	F: CTGATCTCTGCCGTCACCAT	108.7	0.989	89	Beemelmanns et al. [53]
	R: AGAAGACTGATGAGGTGAAGACA				
*Elongation factor* *1 alpha* (*ef1a*) (NM_001141909)^a^	F: GTGGAGACTGGAACCCTGAA	96.7	0.999	155	Jones et al. [57]
	R: CTTGACGGACACGTTCTTGA				
*60S ribosomal protein 32* (*rpl32*) (BT043656)^a^	F: AGGCGGTTTAAGGGTCAGAT	98.8	0.998	119	Xue et al. [58]
	R: TCGAGCTCCTTGATGTTGTG				
*Eukaryotic translation* *initiation factor 3 subunit* *D* (*eif3d*) (GE777139)^b^	F: CTCCTCCTCCTCGTCCTCTT	101.2	0.998	105	Caballero-Solares et al. [55]
	R: GACCCCAACAAGCAAGTGAT				
*Polyadenylate-binding* *protein 1* (*pabpc1*) (EG908498)^b^	F: TGACCGTCTCGGGTTTTTAG	94.9	0.998	108	Caballero-Solares et al. [55]
	R: CCAAGGTGGATGAAGCTGTT				
*RNA polymerase II* (*polr2*) (CA049789)^b^	F: TTCTGAAAGACCCCCAAGTG	90.0	0.993	145	Hixson et al. [56]
	R: AGCTCGCTGATGAGGTCAGT				

^a^Normalizer gene chosen for this study. ^b^Normalizer gene tested, but ultimately not chosen for this study.

**Table 4:** Morphological and production metrics for Atlantic salmon first reared at 12°C and fed the control diet for a period of 16 days, and then after exposed to an incremental temperature increase up to 16°C (at 0.2°C day^−1^).

	12°C		16°C	
	Mean ± SE	*n*	Mean ± SE	*n*
Weight (g)	463.8 ± 3.5^a,1^	450	557.3 ± 5.6^b^	400
Length (cm)	32.6 ± 0.1^a,1^	450	35.1 ± 0.1^b^	400
K	1.33 ± 0.01^b,1^	450	1.28 ± 0.01^a^	400
Weight gain (g)	—	—	92.0 ± 3.5	400
TGC [g^1/3^ (°C d)^−1^]	—	—	0.85 ± 0.03	400
SGR (% body weight day^−1^)	—	—	0.41 ± 0.02	400
Feed intake (% body weight day^−1^)	0.80 ± 0.02^1^	9	0.70 ± 0.03	9
HSI (%)	1.33 ± 0.08	9	1.19 ± 0.06	9
Fillet yield (%)	50.0 ± 1.3	9	52.3 ± 0.9	9

For each parameter, values without a letter in common are significantly different (*p* < 0.05). K: Fulton's condition factor; TGC: thermal growth coefficient; SGR: specific growth rate; HSI: hepatosomatic index. ^1^Weight, length, K, and feed intake at 12°C were measured at the start of the experiment.

**Table 5:** Morphological and production metrics for Atlantic salmon after 65 days fed either the control diet, experimental diet ^#^1 (ED1), or experimental diet ^#^2 (ED2). Fish were fed these diets as temperature was maintained at 16°C for 3 weeks and then raised to 18°C where temperature was held for 5 weeks.

	Control		ED1		ED2	
	Mean ± SE	*n*	Mean ± SE	*n*	Mean ± SE	*n*
Weight (g)	604.3 ± 14.5	126	609.9 ± 15.5	129	605.7 ± 13.3	130
Length (cm)	37.3 ± 0.2	126	37.4 ± 0.2	129	37.2 ± 0.2	130
K	1.13 ± 0.01	126	1.13 ± 0.01	129	1.15 ± 0.01	130
Weight gain (g)	47.4 ± 9.0	126	52.4 ± 10.1	129	51.1 ± 8.1	130
TGC [g^1/3^ (°C d)^−1^]	0.18 ± 0.04	126	0.19 ± 0.04	129	0.19 ± 0.03	130
SGR (% body weight day^−1^)	0.10 ± 0.02	126	0.11 ± 0.03	129	0.11 ± 0.02	130
Feed intake (% body weight day^−1^)	0.40 ± 0.03	3	0.43 ± 0.07	3	0.35 ± 0.04	3
HSI (%)	0.89 ± 0.03	27	1.04 ± 0.07	27	0.99 ± 0.04	26
VSI (%)	8.41 ± 0.16	26	8.47 ± 0.22	26	8.08 ± 0.21	26
Fillet yield (%)	52.8 ± 0.9	9	51.2 ± 1.3	9	50.5 ± 0.9	9

No significant differences (*p* > 0.05) were found for any parameter between dietary groups. K: Fulton's condition factor; TGC: thermal growth coefficient; SGR: specific growth rate; HSI: hepatosomatic index; VSI: viscerosomatic index.

**Table 6:** Morphological and production metrics, and the incremental thermal maximum (IT_Max_) at which the first 50% of Atlantic salmon succumbed to the increasing temperature protocol. Fish were fed either the control diet, experimental diet ^#^1 (ED1), or experimental diet ^#^2 (ED2).

	Control		ED1		ED2	
	Mean ± SE	*n*	Mean ± SE	*n*	Mean ± SE	*n*
IT_Max_ (°C)	23.4 ± 0.1	45	23.3 ± 0.2	47	22.9 ± 0.2	48
Weight (g)	416.2 ± 16.9	45	436.4 ± 17.7	47	422.9 ± 17.0	48
Weight gain/loss (g)	−76.0 ± 7.7	45	−71.2 ± 8.9	47	−78.9 ± 4.3	48
Length (cm)	35.3 ± 0.4	45	35.7 ± 0.4	47	35.4 ± 0.4	48
K	0.93 ± 0.02	45	0.93 ± 0.02	47	0.93 ± 0.02	48
HSI (%)	1.07 ± 0.03	45	1.05 ± 0.04	47	1.05 ± 0.04	48
VSI (%)	7.44 ± 0.30	45	7.31 ± 0.23	47	7.35 ± 0.24	48
RVM (%)	0.073 ± 0.002	45	0.070 ± 0.002	47	0.079 ± 0.004	48
Fillet yield (%)	47.2 ± 0.5	9	44.9 ± 1.3	9	44.8 ± 1.7	9

No significant differences between dietary treatments (*p* > 0.05) were found for any parameter. Weight gain/loss is in comparison to the assessment that was performed at 18°C. K: Fulton's condition factor; HSI: hepatosomatic index; VSI: viscerosomatic index; RVM: relative ventricular mass.

**Table 7:** Morphological parameters for Atlantic salmon that survived to the endpoint of the experiment (i.e., when 50% of each population reached their incremental thermal maximum [IT_Max_]); control (*n* = 44), experimental diet ^#^1 (ED1; *n* = 46), and experimental diet ^#^2 (ED2; *n* = 47).

	Control	ED1	ED2
	Mean ± SE	Mean ± SE	Mean ± SE
Weight (g)	541.7 ± 19.1	535.1 ± 23.5	555.6 ± 18.9
Weight gain/loss (g)	−38.8 ± 7.6	−35.0 ± 5.2	−56.2 ± 5.7
Length (cm)	37.6 ± 0.3	37.5 ± 0.4	37.6 ± 0.4
K	1.00 ± 0.02	0.98 ± 0.02	1.02 ± 0.02

No significant (*p* > 0.05) differences between dietary treatments were found. Weight gain is in comparison to the assessment that was performed at 18°C. K: Fulton's condition factor.

## Data Availability

The data supporting this study's findings are available from the corresponding authors upon request.

## Supplementary Materials

Supplementary Materials Supplemental Figure S1: Feed intake (left *y*-axis, points connected by lines) of female triploid Atlantic salmon exposed to an incremental temperature increase (right *y*-axis, gradient line) and fed either the control diet, experimental diet ^#^1 (ED1) or experimental diet ^#^2. Points on the graph indicate the mean feed intake (± standard error) for each dietary treatment over the previous 5 days.
