# Supplementary material for: Influence of Supplemental Dietary Cholesterol on Growth Performance, Indices of Stress, Fillet Pigmentation, and Upper Thermal Tolerance of Female Triploid Atlantic Salmon (Salmo salar)
Source: Aquac Nutr. 2022 Oct 28;2022:6336060. doi: 10.1155/2022/6336060 (PMC9973203; doi:10.1155/2022/6336060)
Supplement: Supplementary Materials — Supplemental Figure S1: Feed intake (left y-axis, points connected by lines) of female triploid Atlantic salmon exposed to an incremental temperature increase (right y-axis, gradient line) and fed either the control diet, experimental diet #1 (ED1) or experimental diet #2. Points on the graph indicate the mean feed intake (± standard error) for each dietary treatment over the previous 5 days. [file 6336060.f1.pdf]

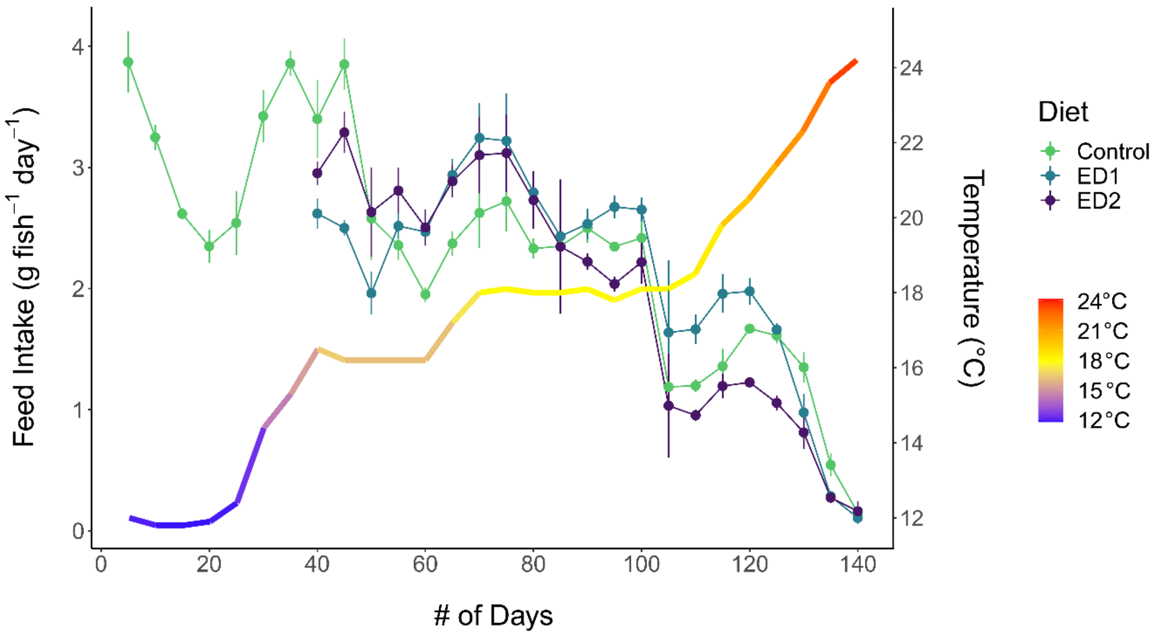

Supplemental Figure S1. Feed intake (left y-axis, points connected by lines) of female triploid Atlantic salmon exposed to an incremental temperature increase (right y-axis, gradient line) and fed either the control diet, experimental diet #1 (ED1) or experimental diet #2. Points on the graph indicate the mean feed intake (± standard error) for each dietary treatment over the previous 5 days.
